# Investigating the Strategies Adopted by Emergency Nurses to Address Uncertainty and Change in the Event of Emerging Infectious Diseases: A Grounded Theory Study

**DOI:** 10.3390/ijerph17072490

**Published:** 2020-04-06

**Authors:** Stanley K.K. Lam, Enid W.Y. Kwong, Maria S.Y. Hung, Wai-tong Chien

**Affiliations:** 1 School of Nursing, Tung Wah College, Kowloon HKG, Hong Kong; mariahung@twc.edu.hk; 2 School of Nursing, The Hong Kong Polytechnic University, Kowloon HKG, Hong Kong; enid.kwong@yahoo.com; 3 The Nethersole School of Nursing, The Chinese University of Hong Kong, New Territories HKG, Hong Kong; wtchien@cuhk.edu.hk

**Keywords:** emerging infectious diseases, emergency nurses, nursing, uncertainty, change, grounded theory, qualitative study, epidemic

## Abstract

Emergency nurses frequently encounter uncertainty and changes during the management of emerging infectious diseases, which challenge their capability to perform their duties in a well-planned and systematic manner. To date, little is known about the coping strategies adopted by emergency nurses in addressing uncertainty and changes during an epidemic event. The present study explored emergency nurses’ behaviours and strategies in handling uncertainty and practice changes during an epidemic event. A qualitative study based on the Straussian grounded theory approach was established. Semi-structured, face-to-face, individual interviews were conducted with 26 emergency nurses for data collection. Adapting protocol to the evolving context of practice was revealed as the core category. Four interplaying subcategories were identified: (1) Completing a comprehensive assessment, (2) continuing education for emerging infectious disease management, (3) incorporating guideline updates and (4) navigating new duties and competencies. The nurses demonstrated the prudence to orientate themselves to an ambiguous work situation and displayed the ability to adapt and embrace changes in their practice and duties. These findings offer insights into the need for education and training schemes that allow emergency nurses to acquire and develop the necessary decision-making and problem-solving skills to handle a public health emergency.

## 1. Introduction

The prevalence of emerging infectious diseases (EIDs) with pandemic potential has increased over the past decades as a result of a boom in global commerce activities and the consequential widespread disruption of ecological systems [1]. Despite the large efforts directed towards advancing strategies and technologies in infection prevention and control, EIDs continue to represent a substantial threat to public health and pose a serious challenge to both developed and developing countries [2]. To address the long-lasting pandemic threats towards public health worldwide and to ensure global health security, it is crucial that international communities and organisations collaborate and coordinate the global endeavours in enhancing public health surveillance and response capacities [3]. 

The capacity of healthcare institutions and facilities to respond to potential outbreaks involves not only implementing contingency plans to meet the precautionary needs but also preparing and equipping frontline healthcare providers for impending EID events [4]. Within the healthcare system, accident and emergency departments (AEDs) are directly accessible to the public and serve as the main gateway for the delivery of healthcare services. Therefore, emergency nurses are often at the forefront of an outbreak response [5]. Indeed, emergency nurses have various duties during the management of an epidemic event, such as the early recognition of suspected or infected patients, implementation of proper infection control measures and coordination of patient logistics [6]. 

Emergency nurses can be confronted with various challenges during EID management. One major challenge faced by emergency nurses amid an EID outbreak is the elevated but unpredictable risk of infection. For example, the infectious status of most of the attendees in the AED is unknown. This uncertainty increases the risk of exposure to frontline emergency nurses compared with that of other healthcare professionals during an epidemic event [7]. In addition to the occupational risks associated with EIDs, a well-reported challenge encountered by emergency nurses is adapting to the changes in the existing guidelines and recommendations for addressing a particular EID [8,9,10]. Studies have highlighted that emergency nurses are required to frequently and rapidly adjust their practice in accordance with amendments to infection control guidelines, even if such amendments are subtle and/or implicit [8,9]. The literature suggests that the antecedents of these challenges could be consequential to the uncertainty and changes experienced by emergency nurses in the workplace during an outbreak: changes and uncertainty within the work environment thus seem to be major and primary barriers to the emergency nursing practice [10].

Although these clinical uncertainties and changes to working practice encountered by emergency nurses have been reported in the literature [11,12], little is known about the strategies that emergency nurses adopt in response to them amid an epidemic. Addressing such gaps might offer insights into the development of plans and interventions for promoting the preparedness and response of emergency nurses in prevailing EID events. The present study aimed to explore the behaviours and strategies adopted by emergency nurses to overcome the challenges of uncertainty and practice changes during an EID event.

## 2. Materials and Methods 

### 2.1. Design

The present study was conducted in Hong Kong during the period of January 2015 to January 2018. A qualitative study was designed to explore the perceptions and experiences of emergency nurses during epidemic events. The grounded theory approach [13] was selected to facilitate the interactive process of data collection and analysis. Grounded theory attempts to understand the involvement of individuals through the exploration and interpretation of their perspectives and meaning within a social phenomenon [14]. This approach prioritises the interpretation of interactions among individuals within the embedded realities of a phenomenon, which offers insight into the elements that shape their beliefs and actions [15]. In addition, this study adopted the Straussian framework of grounded theory data collection and analysis that was developed by Anselm Strauss and Juliet Corbin [13]. The Straussian framework offers explicit and well-defined analytical steps for data analysis with the provision of a coding paradigm. This paradigm facilitates the discovery of associations among conditions, interactions and consequences [13]. The detailed framework of data interpretation supports the procedural operations of the data analysis process, which might help to establish the plausibility and completeness of the findings while preserving the intertwined and dynamic nature of the data [16]. This study thus used the Straussian framework to help gain an in-depth comprehension of the strategies adopted by emergency nurses to address uncertainty and changes in EID management.

### 2.2. Participants

In line with the grounded theory approach, the participants in this study were recruited using a combination of purposive and theoretical sampling strategies. A purposive sampling strategy was used initially to recruit the first 10 participants. This strategy allowed for participants to be selected from the relevant population according to an initial set of criteria [17]. For inclusion, the participants had to be a full-time registered nurse in an emergency department in Hong Kong, Cantonese, English-speaking and willing to participate in the study and share their experiences. After initial recruitment, additional participants were solicited using a theoretical sampling strategy. This sampling strategy uses a systematic and cumulative participant recruitment method and is considered to be the major impetus to the progression of data collection and analysis in grounded theory studies [13]. Theoretical sampling is an iterative process wherein the participant recruitment and data collection processes are driven and performed according to the concepts alluded by the previous data [17]. It enables the ongoing development and elaboration of preliminary concepts and categories that emerge in the data analysis process and thus grants greater representativeness to the findings [18,19]. In general, participant recruitment by theoretical sampling is continued until theoretical saturation is achieved, i.e., when no additional relevant information emerges from the data analysis and the concepts and categories are amply unfolded to display patterns of properties and dimensions [20]. In the present study, the repetition of concepts and categories continued after data had been collected from and analysed for 26 participants, when no additional concepts or categories were yielded. The participant demographic information is summarised in Table 1.

### 2.3. Data Collection

Data were collected from semi-structured, face-to-face, individual interviews held between the participants and the first study author (S.K.K.L.). Once eligible individuals agreed to participate, they were given an information sheet that explained the rationale and objectives of the study. The details of their involvement in the study were also indicated and described in the information sheet. After the participants confirmed that they understood the nature of the study and agreed to participate, an interview was scheduled with each participant at their preferred location and time. Prior to the interview, demographic information of the participant, such as age range, ranking and years of work experience, was collected using a demographic data sheet. The participants gave permission for the interviews to be audiotaped. A total of 26 interviews were conducted and the length of the interviews ranged from 55 minutes to 3 hours. 

### 2.4. Data Analysis

The theoretical sampling, data collection and data analysis processes were performed simultaneously and were thus characterised as a concurrent procedure. The insights gleaned from the interviews during the data analysis helped navigate and inform the directions for subsequent data collection and, consequently, theoretical sampling [15]. Prior to data analysis, each audiotaped interview was transcribed verbatim by the first author (S.K.K.L.). The transcripts were then checked against the interview tapes to ensure the accuracy of the transcription. Once the transcription was completed, data analysis commenced as per the three-phase coding framework suggested by Corbin and Strauss [13], namely open coding, axial coding and selective coding.

The first step of the data analysis process was open coding. The primary goal in this phase was to discover preliminary codes and categories from the data. To start, the textual content of the interviews was read and reread several times by the first author (S.K.K.L.) to capture a general understanding of the participants’ points of view. Each transcript was then scrutinised by examining the content line by line and paragraph by paragraph. The concepts and meaning expressed in each passage that were considered to be of analytical relevance were coded. The codes were further interpreted using a constant comparative method wherein the existing codes were compared with the codes that emerged from the preceding analyses [16]. Codes that showed similarities in features were collated to form categories.

In the axial coding phase, the established categories were refined by examining their connections with one other. In addition to the constant comparative method, the coding paradigm reported by Corbin and Strauss [13] was used as an analytic tool to explore the relationships among categories. This coding paradigm highlights four main components for the establishment of connections among categories, namely phenomena, conditions, interactional strategies and consequences. Related categories were connected and further developed into more sophisticated categories that comprised clusters of categories and subcategories. 

In the selective coding phase, the core category that could underpin the essence of the phenomenon of inquiry was identified. The core category is characterised by several properties, such as frequent appearance in the data, a considerable degree of abstraction, and extensive connections with all other categories and codes [13]. In this study, the determination of the core category was discussed among the authors and a consensus was reached to use Adapting protocol to the evolving context of practice as the core category that could represent the whole phenomenon under study. 

### 2.5. Ethics Approval and Consent to Participate 

Ethical clearance of the study was granted by the first author’s university Human Ethics Committee (no reference number, approved November 2013). Complete information about the nature of the research and participation was provided to participants. All emergency nurses who participated in the study provided written informed consent regarding their involvement in the study and gave permission for their interviews to be audiotaped. Throughout the study, participant anonymity and confidentiality were guaranteed by various strategies, such as removing any personal information and identifiers from the transcripts, masking their identities by replacing their name with unique codes and protecting the digital recordings and documents in encrypted files to prevent unauthorised access.

## 3. Results

**Figure 1:**
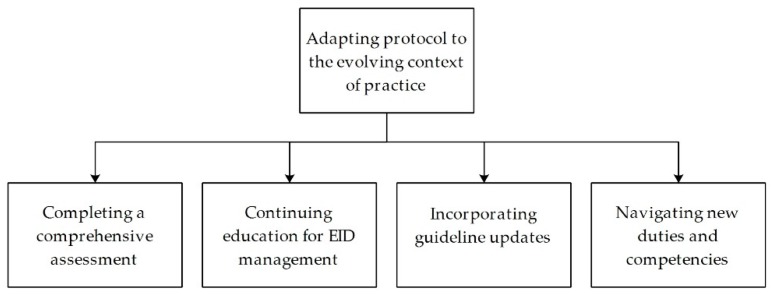
Central categories of emergency nurses’ strategies in addressing uncertainty and change during the management of emerging infectious diseases.

Adapting protocol to the evolving context of practice was identified as the core category that delineates how emergency nurses overcome the uncertainty and change in various areas of their practice during epidemics. Four interplaying subcategories were identified: (1) Completing a comprehensive assessment, (2) continuing education for EID management, (3) incorporating guideline updates and (4) navigating new duties and competencies. These categories represent the strategies adopted by the emergency nurses to address uncertainty and changes during EID management (Figure 1).

### 3.1. Adapting Protocol to the Evolving Context of Practice

Emergency nurses are subjected to a work environment constituting changes and uncertainties in different aspects of emergency care provision during an EID outbreak. To address the diverse needs arising from the evolving context of practice, emergency nurses are required to showcase their capacity to adapt and embrace changes, depending on the situation. The following comment illustrates how an experienced emergency nurse valued the importance of being adaptable when responding to untoward incidents during EID management:

“Various unexpected issues that demand our action come all of a sudden, and we are unable to stop or control them. At this moment, it is time to examine our ability to stand the test of these challenges. It tests our leadership, our problem-solving skills, and our ability to improvise. In addition, it challenges our critical thinking skills and decision-making abilities. These are all crucial as we work in the accident and emergency department, especially in the midst of unpredictable and unforeseen events.”

(P16)

This view was echoed by another participant, who indicated that technical solutions were inadequate and unavailable for emergency nurses to handle unexpected issues while performing EID duties. The participant remarked that it was crucial for emergency nurses to be capable of swiftly adjusting to peculiar situations by identifying alternatives on an impromptu rather than on a prepared basis:

“It could be chaotic and problematic in managing EID. We are not able to predict what is ahead waiting for us to handle. Things can happen in a way that is poles apart from what is written in the protocol.”

(P14)

In addition, the findings suggested that the daily duties of emergency nurses amid an epidemic event were largely affected by the mostly unpredictable individual patient’s circumstances. The uncertainties surrounding a patients’ condition and the nature of outbreak situations posed tremendous challenges to the physical, emotional and psychological capacity of emergency nurses to adapt existing knowledge, skills and attitudes to various circumstances of the patients. One participant shared their experience of an incident that occurred during the Ebola virus disease outbreak. In this situation, a patient with suspected infection displayed uncooperative and aggressive behaviour:

“There was once a suspected Ebola case transferred to our department via ambulance. The patient had developed signs of infection. Therefore, we considered him to be high risk and arranged an isolated room for quarantine purposes. But then the patient started to be uncooperative and aggressive, perhaps because of communication problems, as the patient was from an ethnic minority and there was no interpreter available at that moment. Suddenly he turned violent and assaulted our staff and we had to subdue and restrain him, while we had no time to gown up in PPE (personal protective equipment). At that time, we were so helpless, and we were afraid of being infected. The guidelines and protocols did not mention what we could do in this situation, and we had to count on ourselves.”

(P14)

This incident showcases a situation in which emergency nurses encountered an unpredictable and unexpected event that, as described by some participants, ‘stirred up troubles.’. Instead of following the established protocol, the emergency nurses were required to develop their adaptive capacity to acclimate to the evolving context of practice amid EID management. 

#### 3.1.1. Completing a Comprehensive Assessment

The findings revealed that a major challenge encountered by emergency nurses in managing EIDs was the uncertainty surrounding the patient and disease context. Such an uncertain situation could create ambiguity among emergency nurses in achieving the goals and objectives of their practice. In addition, participants stated that they doubted whether they had been well-prepared for handling an epidemic and questioned the relevance of their prior knowledge and skills in managing EIDs. Some participants expressed the belief that the most pertinent way to resolve uncertainty was to obtain relevant information on how to address any erratic situation. Indeed, gathering up-to-date information was considered crucial by emergency nurses to acquire a general picture of the nature and progress of an EID scenario. This strategy enabled them to comprehensively assess their workplace to orientate themselves to the circumstances. One participant succinctly highlighted the importance of obtaining relevant information when trying gaining familiarity with an EID scenario:

“It is of the utmost importance that you know what is happening. As long as you understand the situation, you realise the problem. You have to acquire the latest information and maintain an up-to-date understanding of the situation.”

(P16)

One of the major concerns raised by the participants surrounded the quality of the information, as some of them pointed out that the information they received was not standardised. Two participants stated that the information provided by their colleagues, which included disease information, infection control guidelines and patient logistics protocols, was sometimes inconsistent, leading to confusion. Although they worked in different hospitals, these two participants held similar opinions about the inconsistency of the information they received. One of the participants described the problem as follows:

“The information could sometimes be regarded as ‘hearsay’. Perhaps one staff member had said something about the disease, then others started to discuss and circulate the information. However, no one had confirmed the creditability or sources of that piece of information. The information might be distorted, exaggerated or even misleading. However, we do not have an official and standardised source for obtaining information, and therefore, hearsay persists among staff.”

(P20)

Many participants highlighted that instead of depending entirely on the provided information, which could be inconsistent, personal alertness and vigilance were also required in addressing the unclear situations they were facing. In their everyday work, emergency nurses serve as gatekeepers who are closely connected to the community. Their frontline position helps emergency nurses to collect clues on disease trends and progression and perform a comprehensive and first-hand assessment of the general disease situation. The comment below illustrates how one participant recognised the outbreak of H1N1 influenza by engaging in routine practice:

“You know about the disease situation and progress at work, especially if you are the triage nurse. There were a large number of patients attending AED and eight out of 10 had similar flu-like symptoms. You would then realise and be able to tell, there was something wrong, it was the influenza that was causing this — you experienced it and sensed it. This sense did not merely improve your alertness, but also provided you with the whole picture of the outbreak, including the severity, the magnitude and the extent.”

(P17)

#### 3.1.2. Continuing Education for EID Management

To respond to an epidemic event, the participants highlighted the importance of acquiring relevant knowledge and skills to bolster their preparedness both theoretically and practically. Indeed, EID management requires emergency nurses to demonstrate proficiency in various skills and techniques. Several participants reported that specific skill sets, such as clinical assessment skills and precautionary measures, enabled them to accomplish various unforeseeable tasks in an effective and appropriate manner during EID management. One advanced practice nurse highlighted the necessity for emergency nurses to develop the skill of rapid and accurate clinical assessment for patient surveillance:

“Sometimes there are junior colleagues making mistakes in simple tasks while handling EID cases. The main reason is that they are not familiar with this type of knowledge. I often ask them to do some infectious diseases revision. This is basic for emergency nurses. For example, being able to identify the signs and symptoms of an EID is the most important task in EID management, but if the nurse did not have the related knowledge, how can one differentiate infected patients from the others?”

(P17)

Because of the importance of obtaining pertinent knowledge and skills, various resources, such as workshops and drills, are available to emergency nurses to facilitate learning on the required techniques to optimally engage in EID management. The participants described that such training courses offered opportunities to AED staff to familiarise themselves with the process of managing epidemic events that were likely to occur. One participant shared their experience of an Ebola drill as follows:

“There was an Ebola drill in our department not long ago. We participated in a simulation for the admission of a patient with confirmed Ebola infection into the AED. It started from the very beginning, from receiving a phone call from the ambulance for the admission of the Ebola case, to triage, to the treatment and arrangement. Staff were assigned to different roles in the drill, such as triage nurse and nursing officer in-charge. We can learn and reinforce the importance of infection control through the drill. We will know how to manage this kind of contingency if we really encounter such an issue in the future.”

(P16)

Indeed, participants agreed that training in a simulated environment assisted their preparation for an epidemic event and enhanced their performance to respond to an EID scenario. Nonetheless, the participants highlighted that it was also necessary for emergency nurses to accumulate clinical experience to establish their preparedness and proficiency in EID management. They described such experience as irreplaceable and stated that full proficiency would not be possible by only participating in drills and education. The participants also stated that prolonged engagement in clinical settings benefitted emergency nurses, as clinical wisdom can only be developed by being immersed in the substantial aspects of everyday practice. The following comment illustrates one participant’s opinion on the value of accumulated clinical experience on establishing preparedness in managing an unanticipated situation:

“In the management of EIDs, there are enormous unexpected issues that one might have no idea about how to handle, unless one had accumulated clinical experience or there were others who could share their own experience. It would be difficult for a junior emergency nurse, who had never come across similar issues in reality, to figure out a solution, even if they had attended workshops or drills before. Only if they had actually experienced this type of issue before, could they identify the possible difficulties that might emerge. Then they would have learned from the experience and be ready to handle similar situations in the future.”

(P22)

#### 3.1.3. Incorporating Guideline Updates

The development of an EID situation could have enormous effects on the implementation of precautionary measures. Although stringent infection control recommendations are solely established to address the exacerbation of the disease situation, the participants valued the relevance of these accelerated infection control measures in protecting both healthcare personnel and the public from EIDs. However, guideline changes for an EID situation also impact emergency service delivery. Some participants did not feel confident about their readiness to adhere to guideline changes due to lack of practice, even though instructions were provided. They commented that there were often distinct differences between new recommendations and the practices they had been accustomed to following, which caused additional difficulties in successfully implementing the new recommendations. One participant expressed concerns about adhering to updated PPE guidelines in an Ebola response and depicted an example of the challenges that emergency nurses might encounter due to guideline changes:

“There are new recommendations on the standard of the PPE kits for use in handling patients with suspected or confirmed Ebola infection, including a new gown, thicker gloves, additional rain boots, and an extra hood. It is different from the one we are used to using when dealing with other infectious diseases. Not only the equipment but also the methods and sequences in equipping and removing the gear are totally new to us. Although we have been told and taught in workshops how to utilise the new PPE, it is still difficult for us to be readily familiar with the new recommendations without an opportunity to practice.”

(P17)

To incorporate guideline updates into practice, participants had underlined the necessity for taking into account the reality of clinical situations and making practical adjustments to new protocol and recommendations accordingly. The following comment gives an example on how emergency nurses might experience the adjustment process:

“Learning from new guidelines and recommendations is like peeling an onion — you peel off something layer by layer. What is left behind is something that could be incorporated into clinical practice. We should understand that what we have learned or adopted formerly is valuable and what we are doing now is renewing it to fit the situation. We do not have to do everything by the book. Instead, we should adopt the process and adapt to the situation.”

(P26)

Indeed, many participants suggested that it was of paramount importance to value both their experience and the new recommendations through careful adjustments and modifications. Emergency nurses might develop a tailored set of practices by integrating new recommendations into their experience, enabling them to adapt to the novel needs of EID management. One participant illustrated their experience in adapting to changes in routine as follows:

“When handling a new situation and coping with new challenges, your experience is always invaluable. However, it is important to understand the core intentions embedded in the new guidelines and recommendations and combine what I have learnt with the new knowledge. I would filter the new guidelines and incorporate them alongside my original set of practices, as long as those guidelines do not violate the basic principles of what I have established from my clinical experience. It is the essence of the new guidelines that I should take into account instead of rigidly adhering to any recommendations.”

(P14)

#### 3.1.4. Navigating the New Duties and Competencies 

During an EID event, the scope of emergency healthcare services is broadened such that emphasis placed on infection prevention and control, in addition to the usual life-saving practice of emergency care provision. Although all participants acknowledged the participation of emergency nurses in an epidemic event response, some encountered difficulties incorporating their extended duties into practice. Several participants commented that performing the extended range of responsibilities was challenging because of a lack of clarity surrounding their scope of practice during an epidemic event. For instance, one participant, who was relatively new to the emergency care setting, expressed the following concerns about performing the responsibilities of an emergency nurse during an H7N9 avian influenza epidemic:

“In the course of EID management, I have a feeling that I am not working in an AED. I formerly expected that an emergency nurse was responsible for triaging patients according to their conditions and offering care to those who were in critical and urgent need. But now my duty has changed all of a sudden and I am mainly assigned to duties on infection prevention. It is true that the guidelines and measures for disease prevention and infection control have been put in place, but the problem is, I personally am not yet in place.”

(P12)

On the same topic, a participant who served as a department operations manager and was in a high managerial position highlighted the importance of emergency nurses to maintain an open mind in the face of significant changes to work practices. This participant addressed their views as follows:

“Ideally, we should do everything we can for the patients, and this is what we always do in AEDs. At the same time, we should also consider the outcomes and consequences of our actions and interventions. There are various issues we have to consider when making a decision, and therefore, we have to be flexible and remain open on these grounds rather than sticking to the same old rut all the time.”

(P26)

Some participants agreed that working adaptively and flexibly was essential for emergency nurses to navigate their altered scope of practice to accommodate the evolving needs during EID management. They generally acknowledged and displayed an acceptance of the extended responsibilities and endeavoured to cultivate adaptiveness and assimilate infection prevention into their regular work practices. One participant commented on this point as follows:

“Sometimes one should allow changes to take place and show openness towards the changes. Now, I can say the practice of infection control has seemingly integrated as a usual component into my emergency care practice, regardless of the alert level and disease situation. It is a process that takes time.”

(P13)

## 4. Discussion

The emergency nurses in this study demonstrated the prudence to orientate themselves to an ambiguous work situation and displayed the flexibility to embrace changes in their routine and practice. According to the findings, the uncertainties surrounding the workplace environment amid epidemic-created obstacles for emergency nurses, preventing them from performing and adopting the skills and tactics required to handle EID management tasks in a fully prepared manner. To address this problem, emergency nurses attempt to obtain information regarding an encountered situation, which involves information about the disease and specific guidelines in response. Acquiring precise information during an EID event is essential for nurses as it provides them with the relevant facts, such as disease identification, management and prevention. In addition, effective information provision could strengthen nurses’ capacity to offer health promotion and health education in the community, which could help calm public fears about EIDs [21]. Despite the importance of the swift provision of epidemic information, the emergency nurses included in this study noted that the disease information they receive might be temporary or incorrect, and such erroneous information can lead to confusion and conflict in practice. In fact, similar problems regarding issuing unreliable information during epidemics have been reported in previous studies [22,23]. These findings indicate the need for healthcare facility administration and management to review and revise the effectiveness of current information dissemination strategies and systems. The findings of this study suggest that healthcare administrators should not merely disseminate information among nurses across services but should also appropriately streamline information to facilitate its integration into routine practice.

In addition to obtaining information from official sources, the emergency nurses in this study reported that they often gain an overall impression of a disease situation through observation and clinical encounters, i.e., by evaluating a disease situation in terms of the number of patients presenting with similar symptoms and the severity of the disease. Although this strategy is seemingly useful to nurses for obtaining first-hand information on an EID event, it may result in inaccurate estimations of the disease situation and adversely affect their awareness of the situation [24]. For instance, Sridhar et al. [25] reported that healthcare workers might underestimate the likelihood of Ebola infection because its incidence and seriousness are comparatively lower based on their practice experience than those quoted in the existing data. Consequently, this underestimation might undermine their awareness of the disease. The findings of the present study underpin the importance of maintaining effective communication between healthcare facilities and frontline healthcare workers, particularly emergency nurses, in improving the estimation of the magnitude of an EID situation.

Apart from the uncertainties surrounding the workplace amid epidemics, the participants of this study considered changes as another major barrier to fulfilling their duties. Such changes include changes in the disease situation, in the information provided and in emergency nursing practice. As frequently reported in the literature, changes in the workplace typically create tension for an organisation’s stakeholders, including those who decide to initiate the changes and those who are required to implement the changes [26]. Changes in the general clinical context of EID management can have a considerable impact on the usual practice, expectations and work practices of the nurses. For instance, a change of the disease situation might induce structural changes in the workplace, which, in turn, might require nurses to change a well-adapted and accepted behaviour or working style to a new and unfamiliar practice. These changes might induce insecurity and create further uncertainty among nurses [27]. Other workplace changes might pose challenges to nurses’ practice. For example, an increase in workload might increase the likelihood of mistakes being made during practice [28]. This issue may partially explain the reluctance of some of the participants included in the present study to accept changes made in different aspects of emergency care provision during EID management.

Our findings highlight that some emergency nurses exhibit a willingness to adapt to changes despite the possible difficulties as they realise the importance of those changes in addressing the new EID management challenges. However, some participants stated that the time and support provided to frontline emergency nurses to adjust their routine and incorporate the changes into their practice was insufficient. This finding is in line with those of previous studies, showing that changes made within healthcare facilities might not always be in line with the ability of healthcare workers to adjust [29,30]. Discrepancies may exist between the expectations of hospital administration on the renewal of existing practices and the actual preparedness and capacity of the staff to adopt the new practices [8]. We propose that a prudent approach that is sensitive to the overall preparedness of nurses in learning new practices or standards is implemented by healthcare facility administrators to ensure that nurses are adequately trained.

Although key knowledge and skills in public health and infection control are integrated as a compulsory part of most current nursing curricula [31], the findings of the present study indicate that emergency nurses are frequently assigned new duties and are required to perform unfamiliar tasks during an epidemic event. These altered duties and new tasks are often considered by emergency nurses to be far beyond their originally perceived scope of practice. For instance, nurses might be required to shoulder the responsibility of public health surveillance during an EID event, including case ascertainment and contact tracing, which could be perceived by emergency nurses as an extra duty outside of their usual domain of practice. To strengthen the capability of emergency nurses in subsequent epidemics, education and training should be provided to equip them with the relevant skills, knowledge and attitudes required to effectively perform their duties in the unprecedented circumstances of an EID outbreak. The training and education that is provided in hospitals to prepare emergency nurses for EID management is often focused on instilling the technical knowledge and skills required to implement infection control measures, such as hand hygiene practices or PPE use [32]. The provision of training and practice in the acquisition and augmentation of decision-making and problem-solving abilities may be overlooked. Thus, in addition to technical skills, we propose that education and training should place equal emphasis on developing nurses’ cognitive skills, such as critical thinking. Such training will help equip nurses with the core skills required to process and apply knowledge in chaotic and complicated conditions.

It must be noted that the scope of the present study could be limited by the relatively small sample size. Although the sample size was determined by the achievement of data saturation and considered sufficient for the present study, the small number of participants could impose limits on the representativeness of the findings. This would hamper the potential to generalise the results of the present study to the wider study population. Despite the small sample size, the present study has sought to capture some of the complexity attached to emergency nurses’ behaviours and strategies in addressing uncertainty and change amid epidemic events. It is anticipated that the findings of the present study could offer useful insights for future implications on emergency nurses’ preparedness and competence in public health responses.

## 5. Conclusions

This study found that emergency nurses are required to adapt and adjust to the evolving context of practice during an epidemic event. In addition to factual information, emergency nurses are often required to gather first-hand information through everyday practice to assist them in comprehensively assessing the situations they encounter. When addressing their duties and responsibilities, it is important for emergency nurses to demonstrate critical thinking, flexibility and adaptability. To reinforce the preparedness of emergency nurses, learning by practical experience, which preserves the essence of clinical wisdom, should be taken into account as it is an efficient approach to train emergency nurses in EID management.

## Figures and Tables

**Table 1:** Demographic information of the participants.

Participant	Gender	Age Range	Ranking	Years of Nursing Experience
P1	M	25–30	RN ^1^	7
P2	M	25–30	RN	7
P3	M	25–30	RN	2
P4	F	30–35	RN	9
P5	F	25–30	RN	5
P6	M	45–50	NO ^2^	20
P7	F	20–25	RN	1
P8	F	30–35	RN	9
P9	M	20–25	RN	1
P10	F	25–30	RN	6
P11	M	20–25	RN	3
P12	M	20–25	RN	3
P13	M	30–35	RN	15
P14	M	30–35	APN ^3^	12
P15	M	25–30	RN	5
P16	M	45–50	NO	20
P17	F	35–40	RN	15
P18	F	30–35	RN	10
P19	F	35–40	RN	10
P20	M	35–40	RN	15
P21	F	35–40	APN	15
P22	F	25–30	RN	6
P23	F	45–50	WM ^4^	25
P24	F	35–40	RN	15
P25	F	35–40	RN	9
P26	F	50–55	DOM ^5^	30

^1^ RN = Registered Nurse; ^2^ NO = Nursing Officer; ^3^ APN = Advanced Practice Nurse; ^4^ WM = Ward Manager; ^5^ DOM = Department Operations Manager.

## References

[1] Hoberg E.P., Brooks D.R. (2015). Evolution in action: Climate change, biodiversity dynamics and emerging infectious disease. Phil. Trans. R. Soc. B.

[2] Rappuoli R., Black S., Bloom D.E. (2019). Vaccines and global health: In search of a sustainable model for vaccine development and delivery. Sci. Transl. Med..

[3] Lam S.K.K., Kwong E.W.Y., Hung M.S.Y., Pang S.M.C., Chiang V.C.L. (2018). Nurses’ preparedness for infectious diseases outbreaks: A literature review and narrative synthesis of qualitative evidence. J. Clin. Nurs..

[4] Labrague L.J., Hammad K., Gloe D.S., McEnroe-Petitte D.M., Fronda D.C., Obeidat A.A., Mirafuentes E.C. (2018). Disaster preparedness among nurses: A systematic review of literature. Int. Nurs. Rev..

[5] Baduge M.S.P., Morphet J., Moss C. (2018). Emergency nurses’ and department preparedness for an Ebola outbreak: A (narrative) literature review. Int. Emerg. Nurs..

[6] Lam S.K.K., Kwong E.W.Y., Hung M.S.Y., Pang S.M.C., Chien W.T. (2019). A qualitative descriptive study of the contextual factors influencing the practice of emergency nurses in managing emerging infectious diseases. Int. J. Qual. Stud. Health Well-Being.

[7] Choi J.S., Kim J.S. (2018). Factors influencing emergency nurses’ ethical problems during the outbreak of MERS-CoV. Nurs. Ethics..

[8] Lam S.K.K., Kwong E.W.Y., Hung M.S.Y., Pang S.M.C. (2016). Bridging the gap between guidelines and practice in the management of emerging infectious diseases: A qualitative study of emergency nurses. J. Clin. Nurs..

[9] Zimmerman P.A., Mason M., Elder E. (2016). A healthy degree of suspicion: A discussion of the implementation of transmission based precautions in the emergency department. Australas. Emerg. Nurs. J..

[10] Lam S.K.K., Kwong E.W.Y., Hung M.S.Y., Pang S.M.C., Chien W.T. (2019). Emergency nurses’ perceptions of their roles and practices during epidemics: A qualitative study. Br. J. Nurs..

[11] Delgado C., Upton D., Ranse K., Furness T., Foster K. (2017). Nurses’ resilience and the emotional labour of nursing work: An integrative review of empirical literature. Int. J. Nurs. Stud..

[12] Wolf L.A., Perhats C., Delao A.M., Clark P.R. (2017). Workplace aggression as cause and effect: Emergency nurses’ experiences of working fatigued. Int. Emerg. Nurs..

[13] Corbin J.M., Strauss A.L. (2015). Basics of Qualitative Research: Techniques and Procedures for Developing Grounded Theory.

[14] Handberg C., Thorne S., Midtgaard J., Nielsen C.V., Lomborg K. (2015). Revisiting symbolic interactionism as a theoretical framework beyond the grounded theory tradition. Qual. Health Res..

[15] Bryant A., Charmaz K. (2019). The SAGE Handbook of Current Developments in Grounded Theory.

[16] Etikan I., Musa S.A., Alkassim R.S. (2016). Comparison of convenience sampling and purposive sampling. AJTAS.

[17] Giles T.M., de Lacey S., Muir-Cochrane E. (2016). Coding, constant comparisons, and core categories. Adv. Nurs. Sci..

[18] Holloway I., Wheeler S. (2017). Qualitative Research in Nursing and Healthcare.

[19] Gentles S.J., Charles C., Ploeg J., McKibbon K. (2015). Sampling in qualitative research: Insights from an overview of the methods literature. Qual. Rep..

[20] Aldiabat K.M., Navenec L. (2018). Data saturation: The mysterious step in grounded theory method. Qual. Rep..

[21] Stirling B.V., Harmston J., Alsobayel H. (2015). An educational programme for nursing college staff and students during a MERS-coronavirus outbreak in Saudi Arabia. BMC Nurs..

[22] Belfroid E., Timen A., van Steenbergen J.E., Huis A., Hulscher M.E. (2017). Which recommendations are considered essential for outbreak preparedness by first responders?. BMC Infect. Dis..

[23] Lam K.K., Hung S.Y. (2013). Perceptions of emergency nurses during the human swine influenza outbreak: A qualitative study. Int. Emerg. Nurs..

[24] Goodband A., Oakley S., Rayner J., Toms J., Brostoff J. (2014). Influenza: Disease, epidemiology and the importance of vaccination uptake by healthcare workers. Prim. Health Care.

[25] Sridhar S., Brouqui P., Fontaine J., Perivier I., Ruscassier P., Gautret P., Regner I. (2016). Risk perceptions of MSF healthcare workers on the recent Ebola epidemic in West Africa. New Microbes New Infect..

[26] Daiker B.L. (2013). Adaptive challenges in medical practices. J. Med. Pract. Manag..

[27] Corazzini K., Twersky J., White H.K., Buhr G.T., McConnell E.S., Weiner M., Colón-Emeric C.S. (2014). Implementing culture change in nursing homes: An adaptive leadership framework. Gerontologist.

[28] MacIntyre C.R., Chughtai A.A., Seale H., Richards G.A., Davidson P.M. (2015). Uncertainty, risk analysis and change for Ebola personal protective equipment guidelines. Int. J. Nurs. Stud..

[29] Maddigan J., Butler M., Davidson J. (2019). Changing nursing practice: Implementation challenges of intentional rounding on three rehabilitation units. Healthc. Manag. Forum.

[30] Sharma N., Herrnschmidt J., Claes V., Bachnick S., De Geest S., Simon M. (2018). Organizational readiness for implementing change in acute care hospitals: An analysis of a cross-Sectional, multicentre study. J. Adv. Nurs..

[31] Clark M., Raffray M., Hendricks K., Gagnon A.J. (2016). Global and public health core competencies for nursing education: A systematic review of essential competencies. Nurse Educ. Today.

[32] Ellingson K., Haas J.P., Aiello A.E., Kusek L., Maragakis L.L., Olmsted R.N., VanAmringe M. (2014). Strategies to prevent healthcare-associated infections through hand hygiene. Infect. Control Hosp. Epidemiol..

